# Fabrication of Anti-glaucoma Nanofibers as Controlled-Release Inserts for Ophthalmic Delivery of Brimonidine Tartrate: *In Vivo* Evaluation in Caprine Eye

**DOI:** 10.34172/apb.2024.025

**Published:** 2024-01-13

**Authors:** Fariba Shaikhi Shoushtari, Mohammadshakib Naghshbandy, Leila Rezaei, Saba Mehrandish, Shahla Mirzaeei

**Affiliations:** ^1^Department of Ophthalmology, Faculty of Medicine, Kermanshah University of Medical Sciences, Kermanshah, Iran.; ^2^Department of Ophthalmology, Kermanshah University of Medical Sciences, Kermanshah, Iran.; ^3^Pharmaceutical Sciences Research Center, Rahesh Daru Novine, Kermanshah 6715847141, Iran.; ^4^Pharmaceutical Sciences Research Center, Health Institute, Kermanshah University of Medical Sciences, Kermanshah, Iran.; ^5^Nano Drug Delivery Research Center, Health Technology Institute, Kermanshah University of Medical Sciences, Kermanshah, Iran.

**Keywords:** Brimonidine tartrate, Caprine, Electrospinning, Glaucoma, Nanofibers, Ophthalmic drug delivery

## Abstract

**Purpose:**

Chronic ailments usually decrease the quality of life due to the requirement for repetitive administration of drugs. Glaucoma is a chronic eye disease occurred because of increased intraocular pressure (IOP). Controlled-release inserts can overcome this challenge by a gradual release of the antiglaucoma drugs. This study aimed to fabricate ocular inserts of brimonidine tartrate (BMD) for the management of glaucoma.

**Methods:**

Different polymers including poly (D, L-lactide), polycaprolactone, cellulose acetate, and Eudragit RL100® were used to develop the BMD-loaded nanofibrous inserts by electrospinning technique. The inserts were characterized. The morphology and drug-polymer compatibility were examined by scanning electron microscopy (SEM), and Fourier-transform infrared (FTIR) spectroscopy and *in vitro* drug release in PBS. The IOP-lowering efficacy and irritancy of optimized formulation were assessed in the caprines.

**Results:**

SEM images demonstrated nanofibers with uniform morphology and a mean diameter<300 nm were fabricated. The nanofibers were high-strength and flexible enough to be placed in the conjunctival sac. FTIR showed drug-polymer compatibility. *In vitro* release study indicated a sustained-release profile of the drug during 6 days for inserts. *In vivo* evaluation indicated that the optimized formulation is capable of maintaining the IOP in a non-glaucomatous range for an extended duration of 6 days. In addition, the formulation was non-irritant to the caprine eye.

**Conclusion:**

Due to the prolonged IOP-lowering efficiency, BMD-loaded nanofibrous inserts can be considered suitable for the controlled release of drugs and thus enhance patient compliance by reducing the frequency of administration.

## Introduction

 Glaucoma is defined as a group of chronic eye disorders distinguished by progressive degeneration of the optic nerve which would eventually lead to irreversible blindness if it is not controlled.^1^ Glaucoma is usually associated with an elevated level of intraocular pressure (IOP); hence, continuous administration of IOP-lowering agents is a common therapeutic protocol in most cases.^2,3^ The prevalence of glaucoma was increased in the last decades and nowadays, it is known as the leading cause of irreversible blindness in the world.^4^ Administration of IOP-lowering agents i.e., α-adrenergic agonists, β-adrenergic blockers, prostaglandin analogs, carbonic anhydrase inhibitors, etc., is the only confirmed therapeutic protocol for the management of glaucoma; although, in some cases, laser trabeculoplasty and surgery are required at the beginning of treatment.^5,6^

 Brimonidine tartrate (BMD) is an anti-glaucoma agent belonging to the α_2_-adrenergic receptor agonist class that lowers the IOP by both decreasing the inflow and increasing the outflow of aqueous humor. Also, an independent neuroprotective effect was observed for this compound.^7^ Due to the partial water-solubility, BMD is rapidly dissolved and eliminated through the nasolacrimal duct; hence, this drug possessed a low intraocular bioavailability.^8^ BMD is normally prescribed as an eye drop solution that requires two- to three times a day administration. This repetitive administration is not accepted by patients who require using the drug for a long duration, and thus it decreases their quality of life.^9,10^ Accordingly, novel drug delivery systems with modified and prolonged release of drug has been introduced recently to enhance topical ocular drug delivery.^11^

 Topical ocular drug delivery is the most convenient route of administration for anti-glaucoma drugs.^12^ This route of administration is known to have the advantages of being targeted, non-invasive, self-administrable, and having fewer side effects compared to systemic forms. However, fast elimination from the surface of the eye and poor intraocular bioavailability, are major challenges of topical preparation.^13^ Consequently, anti-glaucoma drugs should be formulated as novel drug delivery systems with beneficial properties to conquer these obstacles.

 Ocular inserts are the new trends in ocular drug delivery systems as they can deliver the drug to the eye in a controlled manner and reduce the frequency of administration.^14^ These inserts can be developed as electrospun nanofibers with the benefits of being porous, high-strength, flexible, preservative-free, and having a high surface-to-volume ratio.^15^ These systems are versatile in the selection of matrix components as many biocompatible polymers can be electrospun into nanofibers.^16^ Despite the discussed advantages, the literature review revealed that there are only a few studies focused on the design and development of nanofibrous inserts for ocular delivery of BMD. In a similar study, dendrimer nanofibers containing BMD were prepared using polyamidoamine, which indicated a controlled release profile and sui IOP-lowering efficacy.^17^

 The present study is one of the first studies that used four different biocompatible polymers and blends of those polymers to design and develop nanofibrous ocular inserts for topical ocular delivery of BMD. The prepared nanofibers were characterized for morphology, mechanical, and physicochemical characteristics. *In vitro* release study was performed to investigate the release behavior of inserts. In addition, *in vivo* evaluation of IOP-lowering efficacy of optimized formulation was examined in caprine eyes.

## Materials and Methods

###  Materials

 BMD, cellulose acetate (CA, acetyl content 39.8%, Mw = 30,000 g/mol), polycaprolactone (PCL, Mw = 80,000 g/mol), and poly(D,L-lactide) (PLA, Mw = 20,000 g/mol) were purchased from Sigma-Aldrich (Steinheim, Germany). Eudragit^®^ RL100 (EUD) was procured from Evonik Degussa (Darmstadt, Germany). Dichloromethane (DCM), dimethylformamide (DMF), methanol, tryptic soy broth (TSB), fluid thioglycollate medium (FTM), Sabouraud dextrose broth (SDB), sodium dihydrogen phosphate dodecahydrate were purchased from Merck (Darmstadt, Germany). All materials were of analytical grade.

###  Preparation of BMD-loaded nanofibers

 Five different nanofibers were developed using different blends of CA, PCL, PLA, and EUD polymers. Table 1 indices the composition of each formulation. Different polymers with various hydrophilicity were selected to prepare formulations with a diverse range of release profiles and physicochemical characteristics such as flexibility. The rationale behind fabrication of bi-component fibers was the fact that addition of EUD with a medium hydrophilicity supposed to optimize the hydrophilicity profile of extremely hydrophobic PCL fibers and significantly hydrophilic CA fibers. Also, Eudragits are pH-responsive polymers that have been widely used for electrospinning of nanofibers in order to boost the drug-eluting and permeability efficacy through different types of tissue like skin and colon. Hence, EUD has been blended with other polymers to enhance the drug delivery efficacy of formulations.^18,19^ The electrospinning conditions were set based on the previous studies with slight modifications.^20,21^ To prepare BMD-PCL, BMD-CA, and BMD-PLA, PCL, CA, and PLA 10% w/v solutions were independently dissolved in DCM: DMF (7:3 v/v), DCM: DMF (7:3 v/v), and pure DMF solvent systems, respectively. BMD was added to each solution at 10% w/w of the polymer content and the solutions were stirred (300 rpm) for 3 hours, at 25 °C until the complete dissolution of the drug and polymers.

**Table 1:** The composition of electrospinning solutions of different formulations

**Formulation**	**BMD** (% w/w*)	**PCL** **(% w/v)**	**PLA** **(% w/v)**	**CA** **(% w/v)**	**EUD** **(% w/v)**	**Method of Electrospinning**
BMD-PCL	10	10	-	-	-	Single-jet
BMD-PLA	10	-	10	-	-	Single-jet
BMD- CA	10	-	-	10	-	Single-jet
BMD-PCL-EUD	10	10	-	-	10	Double-jet
BMD-CA-EUD	10	-	-	10	10	Double-jet

* Ratio of BMD to polymer content Abbreviations: BMD: brimonidine, PCL: polycaprolactone, PLA: poly (D, L-lactide), CA: cellulose acetate, EUD: Eudragit RL100

 To prepare BMD-PCL-EUD, BMD-CA-EUD nanofibers, EUD was dissolved in methanol at 10% w/v under continuous stirring at 300 rpm and 25 °C; then, BMD was added to the mixture at 10% w/w of the polymer content. The BMD/PCL and BMD/CA solutions were prepared the same as the method defined in the previous paragraph.

 Single-jet electrospinning was performed to fabricate BMD-PCL, BMD-CA, and BMD-PLA nanofibers. The prepared solutions were loaded in the nozzle and ejected from the needle toward a rotating collector (200 rpm) wrapped in an aluminum foil. A voltage of 20 kV was applied between the injector and collector using a high voltage supply (Fanavaran Nano Meghyas, Tehran, Iran). The polymeric solutions were ejected at a rate of 0.5 mL/h, at an injector to collector distance of 20 cm while the nozzle swept in a 10 cm domain. The temperature was kept at 25 °C throughout the whole procedure.

**Figure 1:**
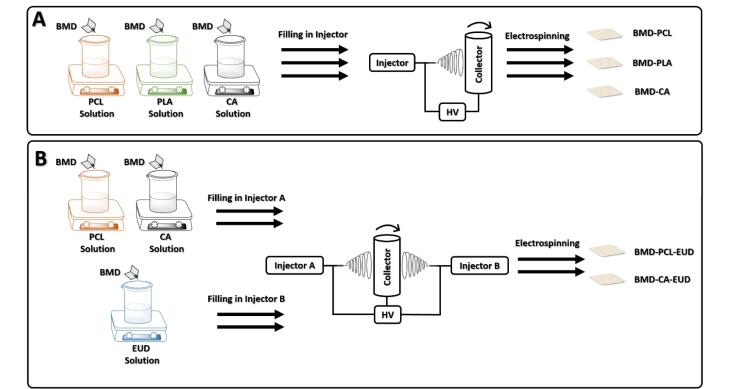
Schematic representation of preparation processes of BMD-PCL, BMD-PLA, BMD-CA (A), BMD-PCL-EUD, and BMD-CA-EUD (B) nanofibrous inserts. (Abbreviations: BMD: brimonidine, PCL: polycaprolactone, PLA: poly (D, L-lactide), CA: cellulose acetate, EUD: Eudragit RL100, HV: high voltage)

 The BMD-PCL-EUD and BMD-CA-EUD nanofibers were electrospun by a double jet electrospinning machine (Fanavaran Nano Meghyas, Tehran, Iran). EUD/BMD solution was loaded in one of the nozzles while the other nozzle was filled with each of the PCL/BMD or CA/BMD solutions. The nozzles were fixed at a frontal position and ejected the polymers concurrently toward the rotary collector at a 0.5 mL/h flow rate. The same electrospinning conditions as described in the previous paragraph were also applied for these formulations. Figure 1 represents the schematic procedure for the preparation of formulations.

###  Scanning electron microscopy (SEM)

 SEM imaging was carried out to characterize the morphology and alignment of the optimized nanofiber (BMD-PCL-EUD). A Piece of nanofiber was coated with a thin layer of gold, then placed in the vacuum chamber of the SU3500 SEM device (Hitachi, Japan) and observed under an accelerating voltage of 20-30 kV.^20^ The obtained image was analyzed by ImageJ software to estimate the mean diameter of fibers and a histogram of diameter distribution was plotted.

###  Fourier-transform infrared (FTIR) spectroscopy

 FTIR spectroscopy is generally performed to detect any influential change in the structure of drug molecules during the preparation process. The BMD, PCL, PLA, CA, EUD, and each of the developed nanofibers were subjected to FTIR spectroscopy. Samples were ground with KBr powder and then compressed into analytical pellets. The FTIR spectra were generated by a spectrophotometer (IR prestige-21, Shimadzu, Japan) at 4000 to 400 cm^-1^. ^20^

###  Thickness and weight uniformity

 The formulations require being uniform across the mat to ensure reproducibility of the results. Pieces of formulations with similar dimensions (2 × 2 cm^2^) were cut from the nanofibrous mat. Weight and thickness were measured by a digital balance and micrometer. An average was taken for each parameter.

###  Entrapment efficiency (EE%)

 The EE% of nanofibers should be calculated to ensure the complete dissolution of drug in the electrospinning solution.^22^ Three samples of each insert were completely dissolved in a proper solvent system and quantified for BMD content by (ultraviolet) UV spectroscopy at a maximum absorbance wavelength of 250 nm. The EE% was measured by equation 1.^21^ A mean value was reported for each formulation.


Eq. (1) $$EE\%=\frac{W_{measured\;drug}}{W_{drug\;used\;for\;preparation\;of\;formulation}}\times 100$$

###  Swelling

 Swelling could be an important factor in the determination of the release behavior of nanofibers. Samples of nanofibers were immersed in 50 mL of distilled water. After 24 hours, the samples were taken out and the surface water was dried by placing them between two sheets of filter paper for 30 seconds. Using the initial and final weight, the degree of swelling was calculated.^21^ The test was repeated three times for each sample and an average was taken.


Eq. (2) $$Swelling\left(\%\right)=\frac{W_{final}-W_{initial}}{W_{initial}}\times 100$$

###  Folding endurance 

 To examine the flexibility and strength of nanofiber folding endurance testing was performed. To evaluate the folding endurance, three samples of each insert were folded repeatedly to 180° until tearing.^20^ The number of times that nanofibers resisted tearing while folding, was recorded as the folding endurance as an indicator of flexibility.

###  Surface pH

 The surface of the inserts was hydrated by placing them in a petri dish containing distilled water for 5 hours under stirring condition.^23^ The surface pH was measured using a pH meter (827 pH lab, Metrohm, Swiss) by placing the electrode on the surface of hydrated inserts. A mean of three readings was calculated.

###  Dry and humid stability

 The nanofibrous insert should poses stability at a range of various relative humidity (RH). To examine the stability of nanofibers, samples of each formulation were cut into similar pieces and weighed accurately. Then, these samples were put in desiccators containing anhydrous calcium chloride and a saturated solution of aluminum chloride to simulate dry and humid conditions, respectively. After 72, the samples were taken out and re-weighed. The moisture loss and uptake percentages were measured using equation 3.^24^


Eq. (3) $$Moistureloss\;and\;uptake\left(\%\right)=\frac{\left|W_{Final}-W_{Initial}\right|}{W_{Initial}}$$

###  In vitro release study

 A bi-chamber model was utilized for *in vitro *evaluation according to the methods used by Mirzaeei et al.^21^ To assemble the donor compartment, the pre-determined weight of each nanofiber was loaded in a cellulose dialysis bag along with 0.5 mL of phosphate -buffered saline (PBS); then the bag was enclosed on both sides. The donor compartment was immersed in 24.5 mL of PBS at a pH of 7.4, as the receptor compartment. The receptor media underwent mild agitation (100 rpm) and the temperature was set at 37 ± 1 °C. Samples were withdrawn at regular intervals from the receptor medium and replaced with an equal volume of fresh PBS immediately to remain at the sink conditions. The released BMD was quantified by UV spectroscopy at a maximum absorbance wavelength of 250 nm.

###  Release mechanism

 The release data were fitted in various kinetic models including zero-order, first-order, Higuchi, and Korsmeyer-Peppas. The correlation coefficient (R^2^) was calculated to determine the best-fitted kinetic model and the release mechanism.

###  Sterility testing

 To ensure the sterility of nanofibers prior to administration of them to animal eyes and to avoid any error in the results by causing an unwanted infection to the eyes of animals, samples of nanofiber were immersed in different culture media to detect any microorganism growth. The samples were exposed to UV radiation for 15 minutes to eliminate any surface contamination. TSB, FTM, and SDB media were utilized for the detection of contamination with aerobic bacteria, anaerobic bacteria, and fungi. For each set of tests, a tube did not receive any samples as the negative control and a tube received a specific microorganism as the positive control. The positive controls were developed by inoculation of *Bacillus subtilis* (ATCC: 21332) in FTM, *Escherichia coli* (ATCC: 25922) in TSB, and *Candida albicans* (PFCC: 62194) in SDB. The culture media were observed at 7-, 14-, 21-, and 28-day intervals.

###  In vivo evaluation of IOP-lowering efficacy and irritancy in caprine eye

 A method used by Mirzaeei et al was utilized with a slight modification to examine the *in vivo *efficiency of the inserts.^21^ Eight Caprines (*Capra aegagrus hircus*) with of 16 glaucomatous eyes (IOP above 13 mm Hg considered abnormal) were subjected to *in vivo *evaluation. It should be noted that animal models were chosen of the animals whose eyes were affected by glaucoma naturally and the elevated IOP was not chemically induced. Pieces (25 mg) of the optimized insert (BMD-PCL-EUD) were administrated in the conjunctival sac of Caprines’ right eyes, while the left eyes received PBS as control. There are reports pointing to systemic absorption of drug following the instillation of eye drop into the eye.^25^ As a result, to avoid affection of the IOP of the eye that received the inserts by the systemic absorption of BMD eye drop instilled in the other eye, PBS was chosen as the control. The IOP changes were recorded using an Air Puff tonometer (Keeler Instruments Inc, Broomall, Pa) within 10 days of administration in both insert and control groups. In addition, the caprine eyes that received the inserts were examined for any sign of irritation or damage including erythema, swelling, abnormal discharge, and corneal opacity during the *in vivo* evaluation.

###  Statistical analysis 

 SPSS software (version 25.00) was used for statistical analysis of results at a significance of 0.05. One-way ANOVA and post hoc Tukey’s tests were performed to compare the physicochemical characteristics of nanofibers. The IOP-lowering efficacy of formulations in animal models was analyzed by Kruskal–Wallis one-way ANOVA and Mann–Whitney U test.

## Results and Discussion

###  Scanning electron microscopy (SEM)

**Figure 2:**
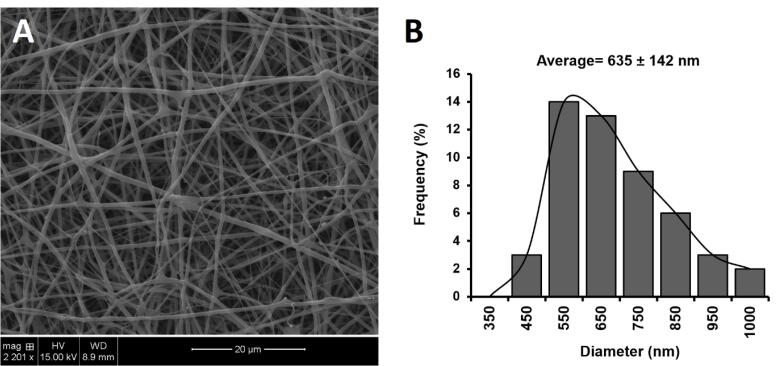
SEM images and the histogram of the size distribution of BMD-PCL-EUD optimized nanofibrous inserts

Figure 2 displays the SEM images and histograms of the size distribution of developed BMD-PCL-EUD nanofiber as the optimized formulation. A uniform morphology with the random alignment of fibers was observed for developed inserts. The formulations showed a mean of 635 ± 142 nm with a normal size distribution through the mat. All formulations indicated a diameter in the sub-micron range which can ensure a high surface-to-volume ratio and thus an enhanced release profile.^26^

###  FTIR spectroscopy

**Figure 3:**
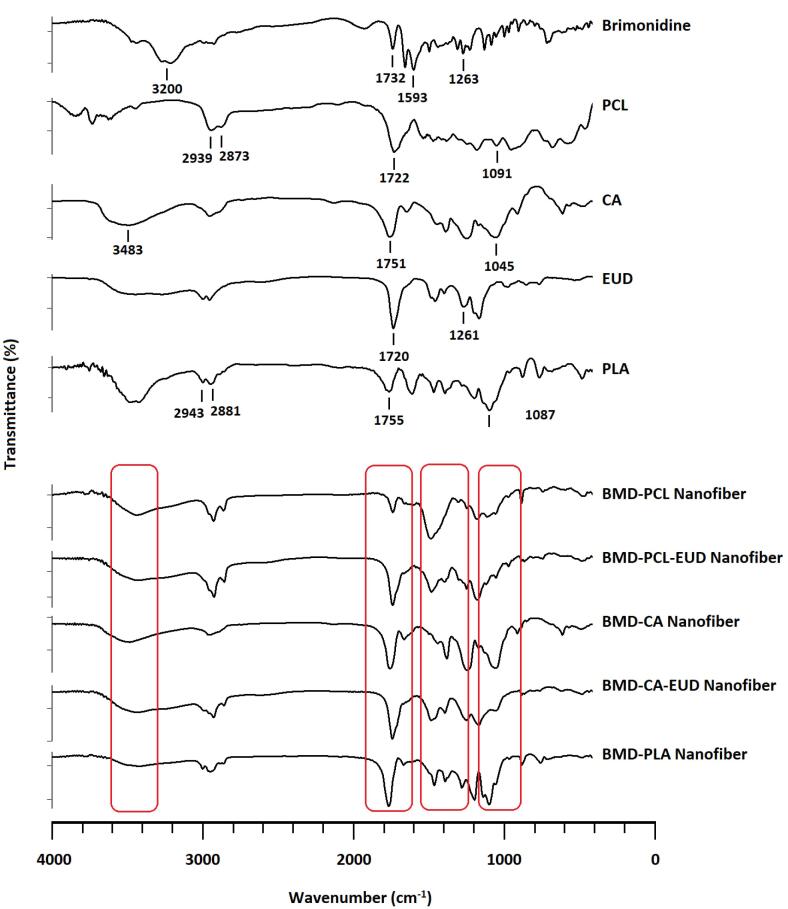
FTIR spectra obtained for BMD, PCL, PLA, CA, EUD, and developed inserts. (Abbreviations: PCL: polycaprolactone, PLA: poly (D, L-lactide), CA: cellulose acetate, EUD: Eudragit RL100)

Figure 3 displays the FTIR spectra of BMD, polymers, and developed nanofibers. FTIR spectrum obtained for pure BMD shows characteristic peaks at 3000-3400 cm^-1^ that are assigned to -NH stretching. Peaks at 1732 and 1593 cm^-1^ are related to C = O and -COOgroups of tartrate salt. A peak at 1263 cm^-1^ is detectable, which is related to -CN stretching.^27,28^ PCL and PLA indicate characteristic peaks at around 2900 and 2800 cm^-1^ that are attributed to asymmetrical and symmetrical CH_2 _stretching. In addition, peaks at almost 1720 and 1090 cm^-1^ are assigned to C = O and C-O-C stretching vibrations of PCL and PLA. Pure CA demonstrates peaks at 3483, 1751, and 1045 cm^-1^ that are respectively assigned to OH, C = O, and C-O-C stretching. In addition, EUD indicates peaks at 1720 and 1246 cm^-1^ owing to the presence of C = O and C-O-C in its structure. All characteristic peaks of BMD appear in FTIR spectra of nanofibers with minor frequency changes that indicate the polymer-drug compatibility.

###  Thickness and weight uniformity

 The formulations indicated weight uniformity with less than 1% of weight changes among pieces. In addition, as represented in Table 2, all formulations indicated thickness uniformity with a mean value of less than 0.300 mm. According to previous studies, thickness values less than 0.400 mm are considered suitable for an ocular insert.^29^ In fact, the formulations are thick enough to preserve their integrity as a prolonged-release insert and thin enough to be non-irritant to the eye.

**Table 2:** The physicochemical characteristics of developed BMD-loaded nanofibers

**Formulation**	**Thickness** **(µm)**	**EE** **(%)**	**Swelling ** **(%)**	**Folding Endurance (times)**	**Surface pH**	**Moisture loss ** **(%)**	**Moisture uptake (%)**
BMD-PCL	197 ± 7	94.3 ± 3.1	150.5 ± 7.1	254 ± 5	6.5 ± 0.4	0.87 ± 0.09	0.97 ± 0.12
BMD-PLA	205 ± 5	97.3 ± 0.9	156.1 ± 3.5	59 ± 1	6.7 ± 0.5	1.23 ± 0.05	1.27 ± 0.21
BMD-CA	198 ± 7	97.6 ± 1.5	195.9 ± 9.5	72 ± 2	6.2 ± 0.5	1.55 ± 0.05	1.84 ± 0.08
BMD-PCL-EUD	220 ± 5	97.9 ± 1.5	187.7 ± 5.3	156 ± 9	5.7 ± 0.6	0.91 ± 0.02	1.02 ± 0.06
BMD-CA-EUD	219 ± 5	94.8 ± 1.6	191.2 ± 6.9	118 ± 2	6.7 ± 0.5	1.05 ± 0.04	1.07 ± 0.04

Abbreviations: BMD: brimonidine, PCL: polycaprolactone, PLA: poly (D, L-lactide), CA: cellulose acetate, EUD: Eudragit RL100, EE: entrapment efficiency.

###  EE%

 According to Table 2, all formulations showed EE% of more than 94% since electrospinning is an efficient method for the fabrication of nanofibers.^30^ It is believed that the high surface area resulting from submicron size is the main reason behind the high entrapment of drug molecules in the electrospun nanofibers. High EE values allow loading of the therapeutic dosing of drug in smaller dosage forms that can eventually decrease the irritancy and increase the patient compliance to self-administration.^31^

###  Swelling

Table 2 indices the degree of swelling obtained for developed nanofibers. The highest degree of swelling belonged to BMD-CA while BMD-PCL possessed the lowest swelling percentage. Generally, due to the hydrophobic nature of PCL and PLA, a lower degree of swelling was observed for the formulations that contained these polymers compared to the ones that contained CA and EUD.^32^ A slightly higher swelling percentage was obtained for BMD-PLA compared to BMD-PCL due to the marginally higher hydrophilicity of PLA than PCL.^33^ Blending EUD with PCL, in BMD-PCL-EUD, enhanced the swelling compared to the formulations containing pure PCL. BMD-PCL-EUD indicated 187.7 ± 5.3% degree of swelling during 24 hours. A similar study reported more than 200% degree of selling for EUD-based nanofibers; it was also noted by this study that although EUD is a water-insoluble polymer, it is classified as a swellable and permeable suitable for sustained drug release.^34^

###  Folding endurance

 A suitable folding endurance ensures that the integrity of inserts was preserved in the conjunctival sac and the formulations are flexible enough to be non-irritant to the eye. BMD-PCL, BMD-PCL-CA, and BMD-CA-EUD formulations showed acceptable flexibility with more than 100 times folding endurance values (Table 2). In a similar study, nanofibers with folding endurance of more than 40 times were considered flexible.^35^ BMD-PCL indicated higher folding endurance compared to other formulations as PCL formed more flexible fibers than PLA, CA, and EUD. A folding endurance value of more than 200 times was observed for PCL-based nanofibers in a similar study.^36^ A similar study reported a higher value of folding endurances for PCL-based nanofibers compared to CA-based nanofibers.^20^ BMD-PLA and BMD-CA indicated a lower level of flexibility by showing a folding endurance lower than 100 times.

###  Surface pH

 As the irritancy and biocompatibility of an ocular insert are related to its surface pH. An ophthalmic preparation needs to possess a pH value within the normal range of tear fluid pH in order to be tolerable and safe.^37,38^ According to Table 2, the formulations indicated pH values in a range between 5.7-6.7, which can be considered suitable for the ocular application.

###  Dry and humid stability

 None of the formulations showed a significant change in weight during three days of incubation under dry and humid conditions. The moisture loss and uptake percentage have not raised above 2% of initial weight for inserts, which indicated stability at different RH%.

###  In vitro release study

**Figure 4:**
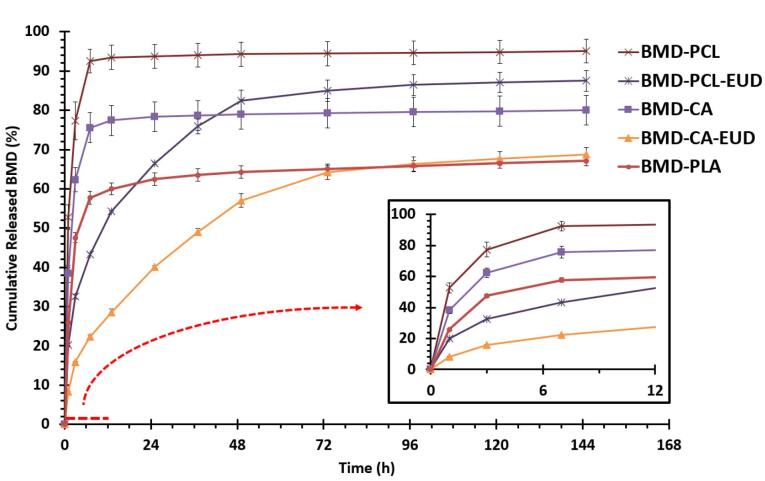
The *in vitro* release profile of brimonidine (BMD) from different nanofibers in PBS (pH = 7.4) at 37 °C with the magnification of first 12 h

Figure 4 represents the results of *in vitro* release study. All formulations indicated a two-phase release profile with a burst release of the drug in the first 7 hours followed by a gradual release during more than 144 hours. BMD-PCL showed a steeper slope during the burst phase indicating a higher rate of release. The lowest rate of release belonged to BMD-CA-EUD. At the end of 24 hours, BMD-PCL, BMD-PCL-EUD, BMD-CA, BMD-PLA, and BMD-CA-EUD released 93.75 ± 3.01%, 66.46 ± 0.35%, 78.34 ± 3.76%, 62.43 ± 1.60%, and 40.06 ± 0.35% of their drug content. It seems that the formulations containing one polymer sustained the release profile to a great extent and extremely slowed down the release rate after 24 hours. Addition of EUD increased the hydrophilicity, so facilitating the drug release reducing the length of plateau phase in BMD-PCL-EUD and BMD-CA-EUD which is more favorable compared to a release plot with a long plateau phase. BMD- PCL-EUD was chosen for *in vivo* evaluation as it showed an appropriate controlled release profile while having high strength and being flexible according to the result of the physicochemical evaluation.

 There are a few studies that developed BMD-loaded nanofibrous inserts, for example, Lancina et al, developed dendrimer-based nanofibers of BMD using polyamidoamine that showed a sustained release of the drug compared to a BMD neat solution.^17^ Other similar studies developed BMD-loaded ocular inserts with film structure. In a study, solvent-casted chitosan films containing BMD were developed that indicated 30-day release of BMD during *in vitro *evaluation.^39^ Also, a 24-hour release of BMD was achieved by Eudragit RSPO-coated film inserts.^40^ Poly(lactic-co-glycolic) acid/polyethylene glycol-based BMD-loaded ocular inserts were fabricated by film-casting in a similar study, which indicated a 1-month release of the drug.^41^ To our knowledge, the present study is one of the first to use four different biocompatible polymers and their blends to design and develop nanofibrous ocular inserts for topical ocular delivery.

###  Release mechanism

 According to Table 3, all formulations showed the highest R^2^ value for the Korsmeyer-Peppas model, except for BMD-CA-EUD that followed the manner of the Higuchi model. Both of these models suggest that drug release from nanofibers is mostly governed by the diffusion phenomenon. BMD-CA-EUD followed the Higuchi manner that indicates that it followed a Fickian diffusion described by equation 4. Where “Q” is the amount of released drug at the time “t”, “A” is the contact area, “C” is the initial drug concentration, “C_s_” is the drug solubility, “D” is the diffusion coefficient, and “K_H_” is Higuchi’s rate constant.

**Table 3:** The R^2^ values obtained by fitting the release data of formulations in different kinetical models

**Formulation**	**Zero-Order**	**First-Order**	**Higuchi**	**Korsmeyer-Peppas**
BMD-PCL	0.3729	0.4908	0.5465	0.7496
BMD-PLA	0.5479	0.6164	0.7247	0.8436
BMD-CA	0.5065	0.5829	0.6900	0.8383
BMD-PCL-EUD	0.9066	0.9880	0.9869	0.9967
BMD-CA-EUD	0.9646	0.9911	0.9981	0.9961

Abbreviations: BMD: brimonidine, PCL: polycaprolactone, PLA: poly (D, L-lactide), CA: cellulose acetate, EUD: Eudragit RL100.


Eq. (4) $$Q=A\sqrt{D\left(2C-C_{s}\right)C_{s}t}=K_{H}\sqrt{t}$$

Korsmeyer-Peppas was the best-fitted model for other formulations described by equation 5, where “M­_t_/M_∞_” is the fraction of released drug at the time “t”, “K” is the release rate constant, and “n” is the release exponent.


Eq. (5) $$M_{t}/M_{\infty }=Kt^{n}$$

In addition, the diffusion exponent (n) was measured to be 0.43, 0.34, 0.38, and 0.55 for BMD-CA, BMD-PCL, BMD-PCL-EUD, and BMD-PLA. Therefore, BMD-CA, BMD-PCL, and BMD-PCL-EUD with n-values lower than 0.45 released their drug content majorly through Fickian diffusion while BMD-PLA with n-value between 0.45-0.89 released their drug through an anomalous transport (non-Fickian diffusion).^42^ Anomalous transport is the characteristic of systems that in addition to diffusion, other mechanisms are involved in the release.^43^

###  Sterility testing

 As all the preparation process was performed in aseptic conditions, the formulations did not show any sign of contamination or microorganism growth in the test tubes during 28 days of sterility test. The insert should be sterile to be administrable in animal studies.

###  In vivo evaluation of IOP-lowering efficacy in caprine eye

**Figure 5:**
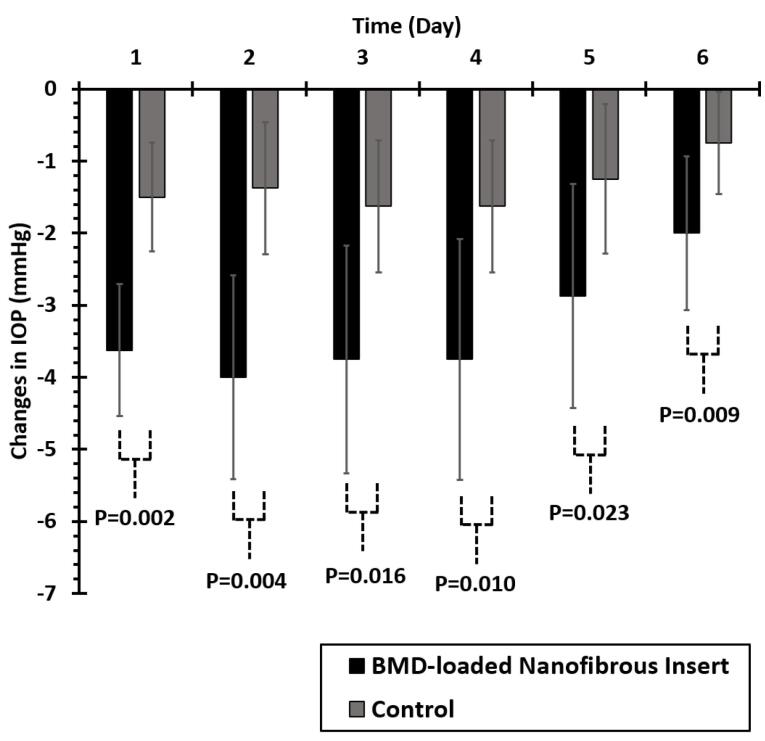
Comparison of mean changes in the Caprine’s eyes intraocular pressure (IOP) after administration of ophthalmic brimonidine (BMD)-loaded insert and placebo (n = 16)

Figure 5 displays the comparison of IOP-lowering efficacy in glaucomatous caprine eyes received BMD-PCL nanofibrous inserts and control. The nanofibrous insert indicated a significantly higher IOP-lowering efficacy for 6 days following the administration in the caprine’s eye. According to previous studies, twice-daily administration of BMD 0.2% w/v eye drop solution would lead to a 3-4 mm Hg IOP-lowering effect.^44^ Hence, the developed insert with a peak IOP-lowering efficacy (-4 mm Hg) on day 2 of administration and more than 3 mm Hg decrease in IOP until day 5, can be considered efficacious compared to conventional eye drop. The prolonged IOP-lowering effect of this formulation can decrease the required frequency of administration to every 5-6 days. Consequently, higher patient compliance is predicted for the developed BMD-loaded nanofibrous insert.

 Both control and intervention eyes were selected from an animal (right and left eyes of one animal). Although control eyes did not receive any medication, a slight reduction of IOP value is detectable in these eyes as well. We believe that this reduction occurred due to the systemic absorption of the drug following topical administration of nanofibers to the other eyes.^45^ Accordingly following the topical administration of BMD-loaded nanofibers to one of the eyes, systemic absorption occurred that caused a slight IOP reduction in the other eye. As Kruskal-Wallis’s test indicated the abnormal distribution of data in intervention and control groups (*P* < 0.05), a non-parametric Mann-Whitney U test was performed to compare the effect of treatment on IOP-lowering efficacy on each day. Significantly higher IOP-lowering efficacy was observed for the intervention groups compared to the control group (*P* < 0.05) during 6 days of examination as represented in Figure 5.

 Although there were not many similar studies, Lancina et al, reported a 2 mm Hg decrease in IOP of rats’ eyes during 6 hours for the developed dendrimer-based nanofibers of BMD.^17^ A prolonged IOP-lowering effect during 8 hours of administration was observed for BMD-loaded film inserts.^40^ Also, in a recent study intravitreal delivery system of BMD was developed that indicated almost 24 weeks of IOP-lowering effect.^46^ In 2022, Zhao et al loaded BMD in sustained-release implants that lowered the IOP for 18 days.^47^

## Conclusion

 Conventional eye drop solutions, usually suffer from a lack of patient acceptance due to the requirement for repetitive administration, especially in the case of chronic eye diseases like glaucoma. The present study aimed to develop brimonidine-loaded nanofibrous inserts for controlled ocular delivery of the drug and achieving a prolonged intraocular pressure lowering effect. Different inserts were prepared to utilize a variety of biocompatible polymers. The inserts indicated a uniform morphology with randomly aligned fibers possessing a mean diameter in the submicron range. Suitable strength and flexibility were observed for the inserts to be placed in the conjunctival sac with neither causing irritancy nor being disintegrated immediately. The FTIR spectroscopy confirmed that no significant change occurred in the structure of the drug and the pharmacologically active moiety while preparation. Additionally, a controlled release of brimonidine within 8 days was observed for inserts during *in vitro* study*. In vivo evaluation* showed non-irritancy of optimized formulation and 4-mmHg decreasing of intraocular pressure during an extended duration of 6 days. The results suggested that a brimonidine-loaded nanofibrous insert with a prolonged effect can be a suitable alternative for conventional eye drops to reduce the frequency of administration and increase patient compliance.

## Acknowledgments

 The authors would like to acknowledge the Research Council of Kermanshah University of Medical Sciences (Grant number: 4010033) for financial support of this work. Also, faithfully thank Rahesh Daru Novin knowledge-based company for cooperation in providing materials and equipment.

## Competing Interests

 There is no conflict of interest.

## Ethical Approval

 In this study, all the experiments were approved by the Institutional Animal Ethics Committee (approval number: IR.KUMS.REC.1400.079), Kermanshah University of Medical Sciences (Kermanshah, Iran).
